# Exploration of *Alicyclobacillus* spp. Genome in Search of Antibiotic Resistance

**DOI:** 10.3390/ijms25158144

**Published:** 2024-07-26

**Authors:** Joanna Bucka-Kolendo, Despoina Eugenia Kiousi, Agnieszka Dekowska, Anna Mikołajczuk-Szczyrba, Dimitrios Marinos Karadedos, Panagiotis Michael, Alex Galanis, Barbara Sokołowska

**Affiliations:** 1 Department of Microbiology, Prof. Waclaw Dabrowski Institute of Agricultural and Food Biotechnology, State Research Institute, Rakowiecka 36 Street, 02-532 Warsaw, Poland; agnieszka.dekowska@ibprs.pl (A.D.); anna.mikolajczuk-szczyrba@ibprs.pl (A.M.-S.); 2 Department of Molecular Biology and Genetics, Faculty of Health Sciences, Democritus University of Thrace, 68100 Alexandroupolis, Greece; dkiousi@mbg.duth.gr (D.E.K.); dimikara91@mbg.duth.gr (D.M.K.); panamich19@mbg.duth.gr (P.M.); agalanis@mbg.duth.gr (A.G.)

**Keywords:** *Alicyclobacillus fastidiosus*, antibiotic susceptibility, antibiotic resistance, horizontal gene transfer, WGS, MIC, AR, HGT, erythromycin resistance

## Abstract

The study investigates the antibiotic resistance (AR) profiles and genetic determinants in three strains of guaiacol-producing *Alicyclobacillus* spp. isolated from orchard soil and pears. Their phenotypic characteristics, such as spore formation; resistance to different factors, including drugs or disinfectants; or production of off-flavor compounds, can affect the taste and aroma of spoiled products. Food and beverages are potential vectors for the transfer of antibiotic resistance genes, which is a growing health concern; thus, microorganisms in food and beverages should not be a potential source of drug resistance to consumers. Whole-genome sequencing (WGS) was utilized to identify antibiotic resistance genes, metabolic pathways, and elements associated with guaiacol and halophenol production. Minimum inhibitory concentration (MIC) testing revealed that all strains were susceptible to eight out of nine tested antibiotics (ampicillin, gentamycin, kanamycin, streptomycin, clindamycin, tetracycline, chloramphenicol, and vancomycin) but exhibited high resistance to erythromycin. Analysis indicated that the erythromycin resistance gene, ribosomal RNA small subunit methyltransferase A (*RsmA*), was intrinsic and likely acquired through horizontal gene transfer (HGT). The comprehensive genomic analysis provides insights into the molecular mechanisms of antibiotic resistance in *Alicyclobacillus* spp., highlighting the potential risk of these bacteria as vectors for antibiotic resistance genes in the food chain. This study expands the understanding of the genetic makeup of these spoilage bacteria and their role in antimicrobial resistance dissemination.

## 1. Introduction

*Alicyclobacillus* spp. are common, spoiling, nonpathogenic, Gram (+), spore-forming, thermo-acidophilic bacteria. They are found in various natural and anthropogenic environments, such as soils, hot springs, mine waters, different fruits, juices, and beverages, as they have the ability to survive in acidic, high temperature, and high-pressure conditions [1,2,3,4,5,6,7,8,9,10] This genus includes species that produce off-flavors and antiseptic-, smoky-, and medicinal-tasting compounds, such as guaiacol and halophenols, 2,6-dibromophenol, and 2,6-dichlorophenol [1,3,11]. Currently, 29 species of *Alicyclobacillus* are known [4,6,11], with 9 of them reported to produce guaiacol: *A. acidophilus*, *A. acidoterrestris*, *A. daucy*, *A. herbarius*, *A. cycloheptanicus*, *A. pomorum*, *A. contaminans*, some strains of *A. hesperidum*, and (as recently reported) *A. fastidiosus* [11].

*Alicyclobacillus* spp. pose a significant concern for the food and beverage industry due to their potential to cause substantial economic losses. Consequently, most studies have focused on identifying and characterizing their growth, germination, behavior, adaptation, metabolite production, and control measures [1,2,3,11,12,13,14,15,16]. Agricultural fields and fertilized orchards may serve as hotspots for the duplication of genes that confer drug resistance, facilitating their transfer to soil bacteria and, subsequently, the food chain. This pathway could allow resistance determinants to reach the human body, making it crucial to uncover potential antibiotic resistance (AR) transmission pathways and the critical mechanisms involved in antibiotic resistance. Limited information is available regarding antibiotic susceptibility among *Alicyclobacillus* spp. Aulitto et al. [2] identified the *brcC* gene involved in bacitracin resistance in two *A. mali* genomes. They also reported that *A. mali* FL18 exhibited resistance to ampicillin, bacitracin, kanamycin, streptomycin, erythromycin, ciprofloxacin, and vancomycin. Based on genomic analysis, they hypothesized that such a broad spectrum of antibiotic resistance is associated with the expression of multiple multidrug efflux transporters. The potential for food and beverages to act as vectors for the transfer of antibiotic resistance genes is a growing health concern [17,18]. The removal of antibiotics from the market in accordance with Regulation (EC) N 1831/2003 has underscored the need to reduce antimicrobial resistance in the food chain [18]. Microorganisms in food and beverages should not be a source of drug resistance to consumers. Resistance to antimicrobial agents can be intrinsic, as natural phenotypic traits typical of all strains of all species, or acquired through additional genes carried on mobile genetic elements (plasmids or transposons) or mutations of indigenous genes [17,19].

One of the key mechanisms for the formation of new genes, including antibiotic resistance genes, is amplification and subsequent mutations, as shown for trimethoprim [20], sulfonamides [21], and β-lactams [22]. Antibiotic resistance predates the 20th-century “antibiotic era”. Prokaryotic antibiotic resistance genes have been found in environments minimally affected by human activity. For instance, the *Sul2–strA–strB* gene cluster was identified in a 1200- to 1400-year-old Antarctic ice core sample [23]; genes conferring resistance to β-lactams, tetracycline, and glycopeptides were found in 30,000-year-old Beringian permafrost sediments [24]. Additionally, aminoglycosides, β-lactams, fluoroquinolones, and macrolide resistance factors were isolated from Tibetan glacial lake sediments and soil [25]. Genes encoding resistance to standard antibiotics in these samples were associated with efflux pumps, distinct from the modern antibiotic resistome. The natural source of AR genes is environmental bacteria, especially Actinomycetota [26,27,28]. Since metabolic pathways for antibiotic synthesis are estimated to be 200 to 500 million years old, antibiotic resistance must be of similar age to counterbalance the antibiotic effects [29,30,31].

Intrinsic resistance presents minimal potential for horizontal spread among microorganisms. In contrast, acquired resistance poses a greater risk for horizontal dissemination, enabling rapid inactivation of selected antibiotics through degradation, export from the cell via efflux systems, or alteration of the antibiotic’s target site [19]. However, the distinction between intrinsic and acquired resistance may be blurred; due to the action of transposases or integrases, resistance factors may transfer between chromosomes to plasmids, and the ability to transmit genes horizontally can be gained or lost. An additional important factor in the evolution and spread of antibiotic resistance is that if the genes associated with antibiotic resistance are located on high-copy-number plasmids, this type of amplification enables faster evolution and increased resistance to the agent. Bacteria in heterogeneous environments can develop antibiotic resistance faster than bacteria living in homogeneous environments, especially when the potential relationship between AR and flagella and chemotaxis can be found through efflux mechanisms, biofilm formation, or mobile genetic elements [32]. Piskovsky et al., 2023, suggest that bacterial motility can facilitate dispersal on the surface, which may lead to greater nutrient availability and potentially higher antibiotic resistance. Bacterial swarming is the best-understood relationship between cell motility and the ability of bacteria to cope with antibiotics. Swarming is a form of group motility on surfaces where cells are particularly resistant to antibiotic stress. Reduced exposure of individual cells to antibiotics, leads to tolerance of antibiotics. In addition to swarming, other connections between bacterial motility and the antibiotic landscape have been reported. In particular, antibiotics have been shown to induce motor responses. However, it is not known what impact they have on the evolution of antibiotic resistance.

The FEEDAP Panel (The Panel on Additives and Products or Substances used in Animal Feed) defines the microbiological breakpoints to distinguish resistant from susceptible strains [17]. Microbiological cut-off values are established by examining the distribution of MIC values of selected antimicrobials in bacterial populations of a single species or genus. These cut-off values are used to evaluate microbial products for the presence of antimicrobial resistance. Genetic investigations are required to further determine the nature of resistance, especially when limited or no information on the MIC distribution within the species is known, as is true of *Alicyclobacillus* spp.

This work aims to deepen the understanding of the genetic determinants associated with antibiotic resistance in three guaiacol-producing *Alicyclobacillus* spp. strains. To achieve this, the three strains were sequenced to annotate relevant regions. Additionally, loci involved in functional characteristics were identified using comprehensive bioinformatic analyses, focusing on the presence of prophage regions, mobile elements, and plasmids. Finally, clusters for the production of metabolites involved in the spoilage phenotype were also recognized, which may be significant for comparative and functional genome analysis within this group of food spoilage bacteria.

## 2. Results and Discussion

### 2.1. Assessment of Antibiotic Resistance

The MIC for 9 antibiotics was performed for three strains, KKP 3000, KKP 3001, and KKP 3002, and the reference strain *Alicyclobacillus fastidiosus* DSM 17978. The antibiotics chosen were based on their diverse mechanisms of action belonging to different classes, such as aminoglycosides (kanamycin, streptomycin, gentamycin), macrolides (erythromycin), chloramphenicol, tetracycline, and clindamycin, which inhibit protein synthesis, as well as β-lactams (ampicillin) and glycopeptides (vancomycin), which inhibit the synthesis of peptidoglycan in Gram (+) bacteria. The assumption was made that the strains should be considered resistant if MICs were >256 μg/mL and sensitive if MICs were <8 μg/mL. 

The profiles of antibiotic resistance for examined strains of *Alicyclobacillus* spp. were very similar to each other. All four strains exhibited significant susceptibility to ampicillin, gentamycin, kanamycin, streptomycin, clindamycin, tetracycline, chloramphenicol, and vancomycin (all values < 1 μg/mL). However, all strains displayed high resistance (256 μg/mL) to erythromycin; even at the maximum applied concentration, no inhibition of the growth of the examined *Alicyclobacillus* strains was observed. The MIC values with the lowest concentration (μg/mL) of an antibiotic that inhibits the growth of a given strain of bacteria are presented in Table 1. These results suggest that *Alicyclobacillus* spp. strains are generally susceptible to a broad spectrum of antibiotics commonly used in clinical and laboratory settings.

A highly important aspect of AR in the environment is that the ecosystem is a complex structure, and the usage of antibiotics in agriculture or horticulture may lead to the dissemination of AR genes. Using animal fertilizers or manure, with possible antibiotic-resistant intestinal bacteria, may result in the introduction of antibiotic resistance genes into the soil, which has the potential for horizontal gene transfer (HGT). Similarly, using recirculated water for irrigation may lead to exposure of soil microorganisms to pharmaceuticals. Exposure of soil to macrolide antibiotics increases the relative abundance of numerous gene targets associated with resistance to macrolides and other antibiotics, as well as mobile genetic elements. This occurred at an exposure dose that was unrealistically high but did not occur at the lower, more realistic exposure dose [33]. Georgakakos et al., 2023 [34] suggested that erythromycin is more mobile in the environment when introduced with manure, which is likely the largest source of agriculturally sourced environmental antibiotics. And since it is particularly resistant to natural environmental degradation, it can increase the influence on the microbial communities within it. 

### 2.2. Genome Characteristics

**Figure 1:**
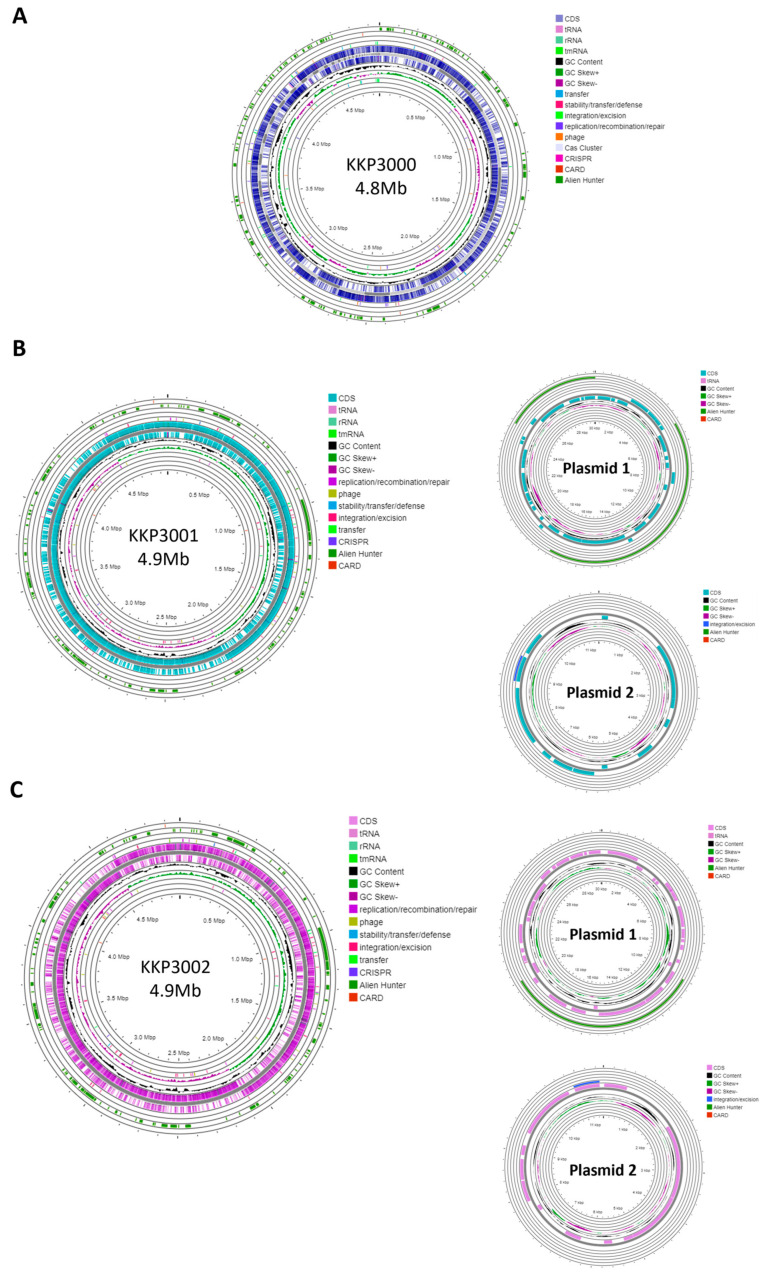
Genomic maps of the chromosome and plasmids of (**A**) KKP 3001, (**B**) KKP 3001, and (**C**) KKP 3002 constructed with Proksee.

Strain KKP 3000 consists of a single chromosome with a length of 4,859,599 bp and a GC content of 52.95% (Table 2). The WGS of strains KKP 3001 and KKP 3002 consists of a chromosome and two plasmids. In greater detail, the chromosome length of KKP 3001 is 4,972,367 bp, and that of KKP 3002 is 4,977,437 bp. The GC content of the chromosomes is 53 and 52.92%, respectively. Strains KKP 3001 and KKP 3002 each carry two plasmids with a size of 30,081 and 11,548 bp, respectively. The chromosome and plasmid maps of the strains are presented in Figure 1.

**Figure 2:**
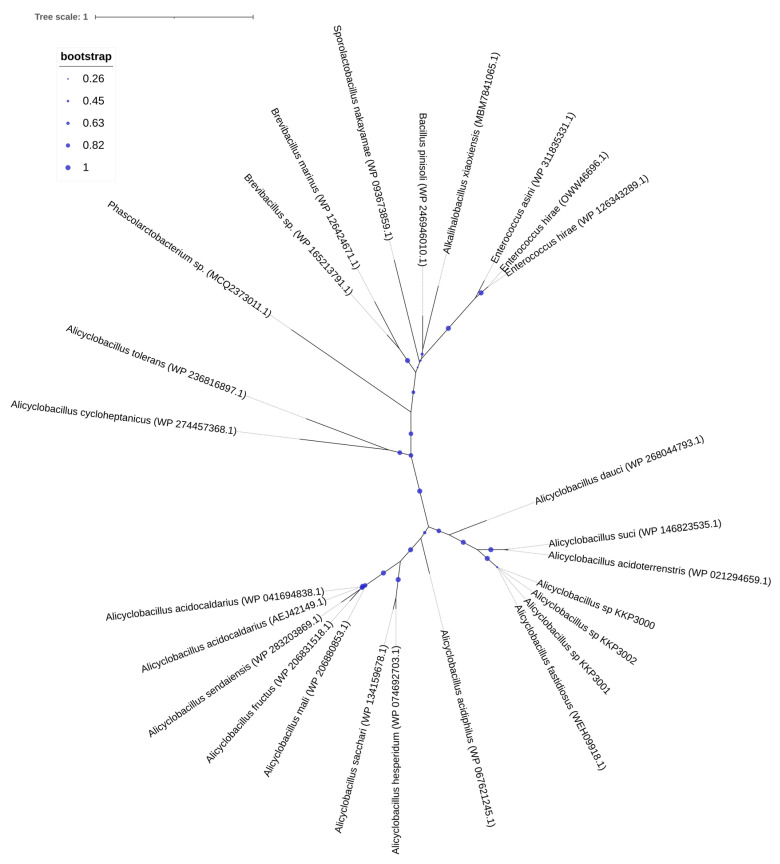
An unrooted tree of homologous RsmA proteins identified and constructed using RAxML and visualized on the iTOL server.

One CRISPR array was identified in each of the strains: KKP 3000, KKP 3001, and KKP 3002 (Table 2, Figure 1). Concerning transposable elements, 52 IS were identified in the genome of KKP 3000 and 14 in the genome of the two other strains (Table S1). Furthermore, 5 incomplete prophages were annotated for KKP 3000 and 7 for KKP 3001 and KKP 3002 (Table S2). AlienHunter was used to pinpoint regions containing mobile elements. To this end, 121, 97, and 98 regions were identified in the genome of KKP 3000, KKP 3001, and KKP 3002, respectively. Accordingly, 76 elements related to mobile element transfer, replication, and integration were identified by mobileOG in the genome of KKP 3000 and 92 in the chromosome of KKP 3001 and of KKP 3002 (Tables S3–S5). Five strict hits (<70% identity) for transferable antibiotic resistance genes were found in the genome of the strains, as shown in Table S6. Specifically, all three strains code for qacG, FosBx1, VanT, and VanW, which may confer resistance to disinfectant agents and antiseptics, as well as glycopeptides and Fosfomycin (Table S6). QacG protein acts as a proton-dependent efflux, and FosBx1 is an Mn+ dependent fosfomycin thiol transferase. The VanT is a membrane-bound serine racemase that converts L-Ser to D-Ser, resulting in D-Ser being used for peptidoglycan synthesis. The VanW protein is possibly involved in antibiotic target alteration, but its mode of action is unknown. In all three analyzed strains downstream of the *vanT* gene, are three genes encoding alanine dehydrogenase, dipeptidase, and permease, which strongly resemble parts of *van* resistance operons found in other bacteria [35]. The CARD database confirmed the alanine dehydrogenase gene as a distant analog of VanH in the *vanF* cluster; the dipeptidase gene found downstream *vanT* gene in analyzed strains does not show any resemblance to known *vanX* genes that encode dipeptidase in vancomycin resistance operons. Additionally, the permease gene mentioned above does not show any resemblance to any known *van* genes. The region encoding part of the putative *van* operon is limited by IS21 upstream and by IS110 downstream and is not functional. Downstream IS110, there are two genes, a sensor histidine kinase and an OmpR superfamily response regulator transcription factor, which functionally resemble *vanS* and *vanR* genes, respectively. At the protein sequence level, the response regulator shows significant similarity to VanR and the kinase to VanS in various clusters. The same two genes were identified in direct proximity to the putative *vanW* gene. It is possible the described genes were parts of the functional *van* operon, which was transferred and disrupted by transposition events. Evidence for the acquisition of these genes through HGT were provided by AlienHunter. Of note, the same HGT region contains the gene *fosBx1*. According to CARD, this gene is chromosomally encoded in *Bacillus* spp. and *Staphylococcus* spp., while mobile fosfomycin resistance genes have been identified in the genomes of clinically relevant *Enterobacteriaceae*, including members of the *Escherichia* and *Salmonella* species [36]. Furthermore, genes coding for ribosomal RNA small subunit methyltransferase A (RsmA) that could confer resistance to erythromycin were identified in the genome of the three strains (KFAPOJEI_01910, PKEAFELD_04479, PLKKBLCC_04475), that were not correlated with mobile or transposable elements. To further investigate the origins of these genes, an unrooted tree of homologous proteins was constructed. As shown in Figure 2, proteins encoded by *Alicyclobacillus* spp. are clustered together possibly indicating that genes are intrinsic and not acquired through HGT, or that they were acquired through a common ancestor in the past. Gene *rsmA* is homologous to *ermC* encoded by *Bacillus* spp. The Erm family of adenine-N(6) methyltransferases confer resistance to macrolide–lincosamide–streptogramin B antibiotics via alteration of their target, as they are responsible for the demethylation of the adenine residue at position 2085 in 23S rRNA interfering with the antibiotic binding [37].

### 2.3. Functional Annotation

The annotated proteins of the three strains were categorized into 19 COGs. As shown in Table 3, the most represented COG in all three strains is amino acid metabolism and transport (class E), followed by transcription (class K) and carbohydrate metabolism and transport (class G). 

**Figure 3:**
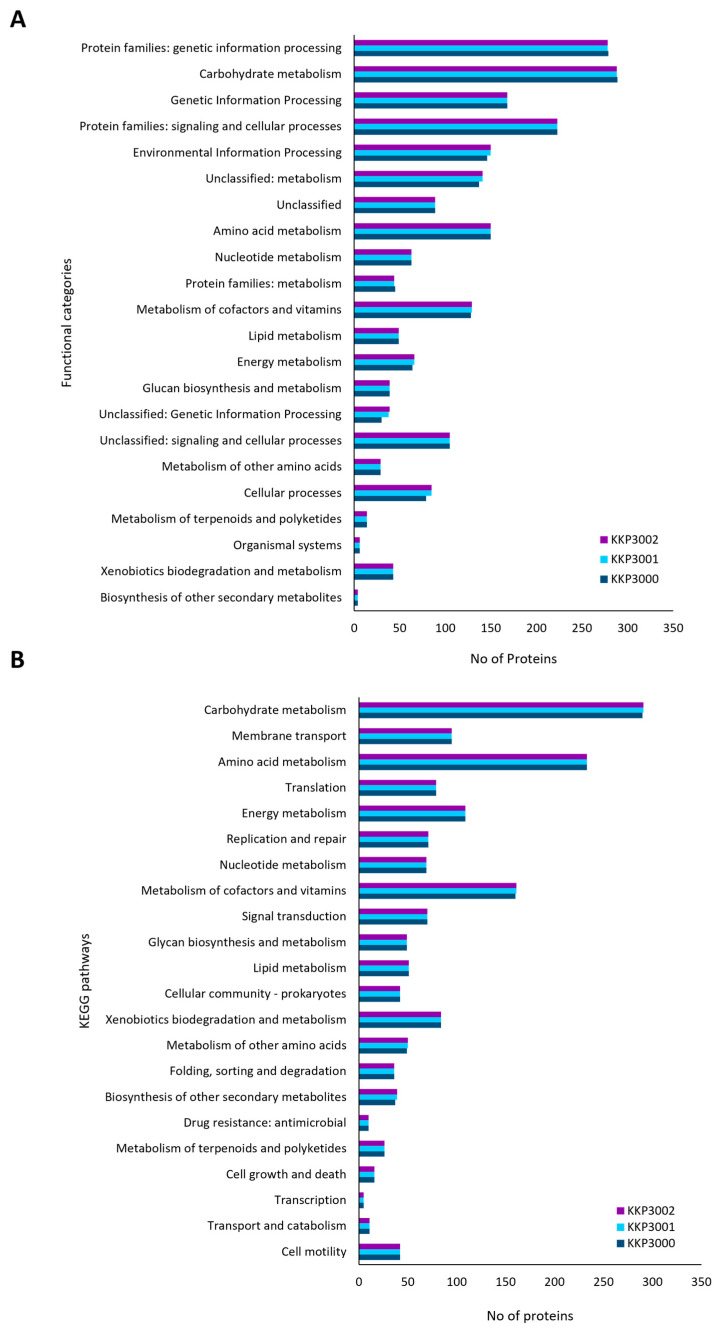
Classification of proteins encoded by KKP 3000, KKP 3001, and KKP 3002 into (**A**) KEGG functional categories and (**B**) KEGG pathways.

Accordingly, annotated proteins were categorized into KEGG functional categories and pathways (Figure 3). The most represented category in all three strains is carbohydrate metabolism, followed by amino acid metabolism and genetic information processing. No significant differences in the distribution of proteins into these categories were noted for the three novel strains.

**Figure 4:**
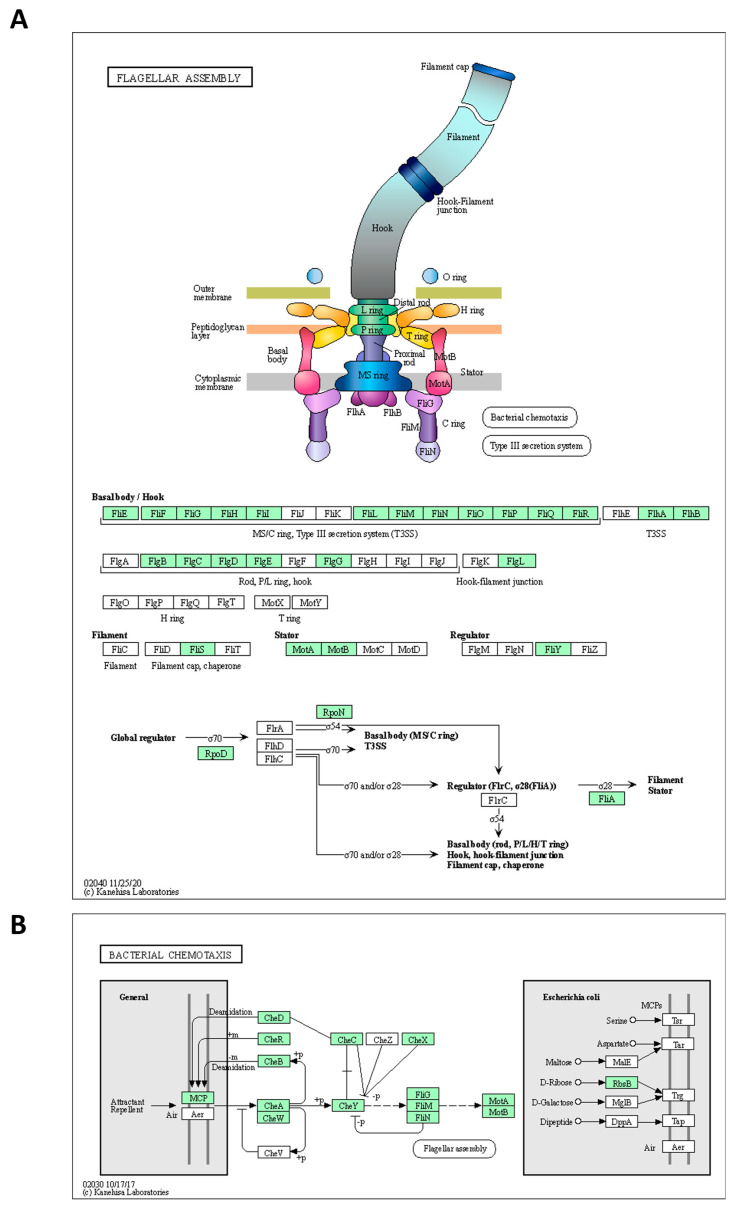
*Alicyclobacillus* strains KKP 3000, KKP 3001, and KKP 3002 code for proteins involved in (**A**) flagellar assembly and (**B**) chemotaxis. The maps were produced using KEGG Mapper. The proteins encoded by the strains are indicated in green.

The genome of the strains was searched for genes involved in spore formation, germination, flagellar assembly, and chemotaxis using annotation algorithms and KEGG modules. The annotated genes are comprehensibly presented in Table 4. In greater detail, 27 genes involved in various processes of sporulation and germination were annotated in the genome of the *Alicyclobacillus* KKP 3000 and KKP 3001 strains. Of note, KKP 3002 does not code for germination protease gpr (Table 4). Furthermore, 41 genes related to flagellar assembly were identified. However, as illustrated by KEGG Mapper (Figure 4), the strains do not code for proteins involved in the formation of the H and T ring, while also missing some important regulatory proteins. On the other hand, the strains code for a full module for bacterial chemotaxis. In this context, flagellar production and swarming behavior were previously recorded for *A. acidoterrestris* and was dependent on pH conditions. Of note, these phenotypes were abolished in low pH, and cells were organized into biofilms to maximize survival [38]. For bacteria, an important adaptive element is the ability to be motile. According to Shemesh et al. [37] bacteria in heterogeneous environments can develop antibiotic resistance faster than bacteria living in homogeneous environments. Since the *Alicyclobacillus* spp. is a species highly correlated with food, it is presumed that motility can facilitate spreading onto surfaces to make nutrients more accessible. Thus, future studies will focus on the investigation of flagellar production and biofilm formation capacity to decipher the ability of the three strains to withstand unfavorable environmental conditions.

### 2.4. Vdc Operons

**Figure 5:**
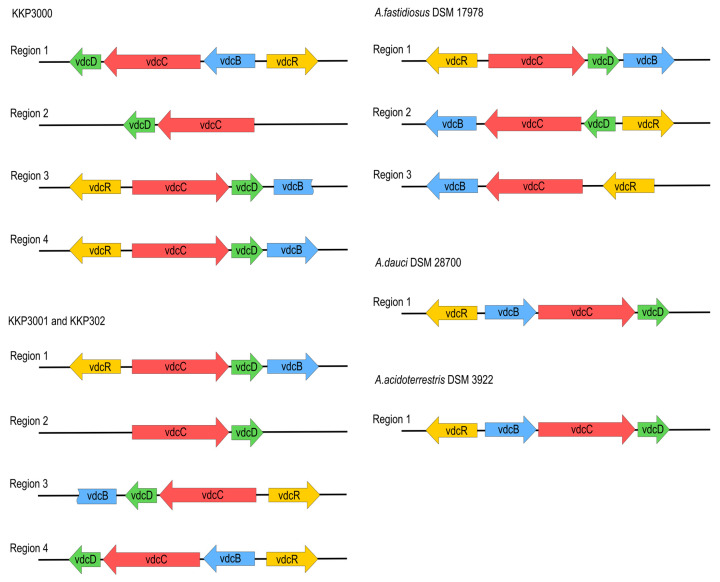
Arrangement of *vdc* operons in novel strains, compared to *A. fastidiosus* DSM17978, *A. acidoterrestris* DSM3922, and *A.dauci* DSM28700.

All analyzed *Alicyclobacillus* strains, KKP 3000, KKP 3001, and KKP 3002 have the ability to produce guaiacol [11]. To this end, proteins associated with the guaiacol production from vanillin were annotated in the chromosomes of the three strains (Table S7). The metabolic pathway for the production of guaiacol from vanillin involves two consecutive enzymatic reactions; the transformation of vanillin to vanillic acid performed by vanillin dehydrogenase, and of vanillic acid to guaiacol by vanillic acid decarboxylase [39]. Local blastp was performed to determine the presence of the genes involved in these enzymatic reactions. All three strains possess the gene coding for benzaldehyde dehydrogenase, the enzyme involved in the first step of the transformation (44% sequence identity and 66% positive substitutions). Accordingly, the strains code for phenolic acid decarboxylase, which is involved in the last step of guaiacol production, with higher sequence identity (78%), and 91% positive substitutions (Table S7). The nucleotide sequences of known *A. acidoterrestris vdc* clusters [11] were compared to genomic sequences of strains, KKP 3000, KKP 3001, and KKP 3002. *A. acidoterrestris* is linked to off-odor production in fruit juices, mainly attributed to guaiacol production in the beverage matrix [39]. The complete *vdc* operon consists of three structural genes, *vdcB*, *vdcC*, and *vdcD*, encoding subunits of non-oxidative vanillic acid decarboxylase Vdc, and a regulator gene *vdcR*, which is located upstream of the structural genes and orientated in the opposite direction [40]. The four regions in each of their genomes related to guaiacol production were found. However, three of them are incomplete or rearranged, probably due to transpositional events. The location and composition of *vdc* regions in all novel strains are similar, especially between KKP 3001 and KKP 3002. To obtain a more complete picture of the *vdc* regions of *Alicyclobacillus*, we also searched for similar regions in complete genome sequences known as guaiacol producers, *A. acidoterrestris* DSM 3922 and *A. dauci* DSM 28700, as well as non-guaiacol producing *A. fastidiosus* DSM 17978 and *A. acidocaldarius* DSM 446. Both guaiacol-producing species carry one complete *vdc* operon in their genomes. We have not found sequences of significant similarity to the *vdc* region in the *A. acidocaldarius* DSM446 genome. *A. fastidiosus* DSM 17978 carries three *vdc*-related regions, although one of them is incomplete, and two others are arranged in a different order than in guaiacol-producing species, which probably is the cause for this strain of *A. fastidiosus* not producing guaiacol. The arrangement of *vdc*-related regions in all analyzed species is shown in Figure 5.

**Figure 6:**
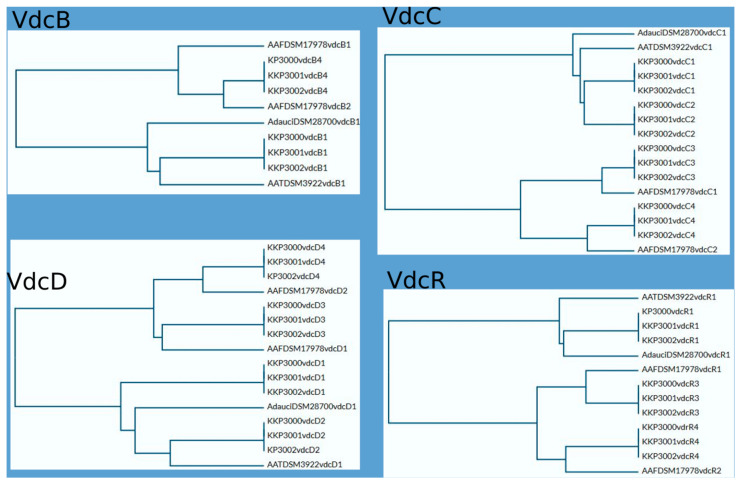
Comparison of Vdc proteins. Phylograms were prepared using UniProt software https://www.uniprot.org/ (accessed on 24 April 2024).

As mentioned above, all novel species share significant similarities regarding locations and composition of *vdc* regions in their genomes. This similarity is particularly evident between KKP 3001 and KKP 3002. Sequence comparison of VdcB, VdcC, VdcD, and VdcR proteins show a higher level of similarity between respective Vdc proteins of particular regions of KKP 3000, KKP 3001, and KKP 3002 than intragenomically, which suggests that divergence of those strains may have occurred later than the duplication of the *vdc* genes. Phylograms based on Vdc proteins sequence alignment are shown in Figure 6.

### 2.5. ANI, and Phylogenomic Analysis

**Figure 7:**
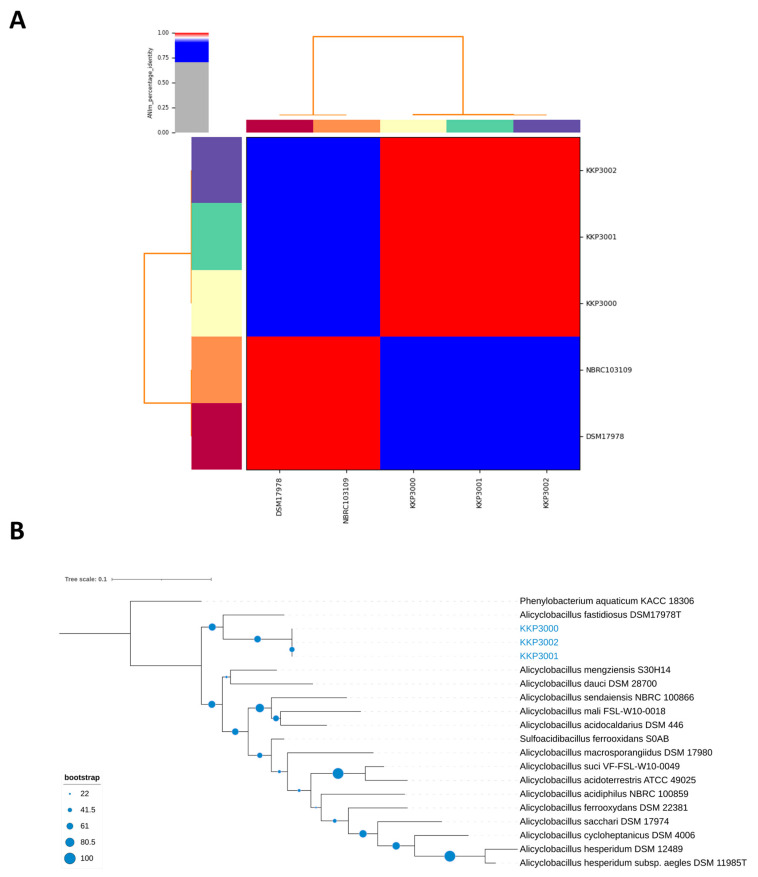
(**A**) ANI of KKP 3000, KKP 3001, and KKP 3002 with members of the *A. fastidiosus* species (i.e., DSM 17978 and NBRC103109). (**B**) The phylogenomic tree containing the three novel strains and members of the *Alicyclobacillus* genus. *Phenylobacterium aquaticum* KACC 18306 was used as an outgroup. The tree was calculated on the TYGS server and visualized using iTOL.

**Figure 8:**
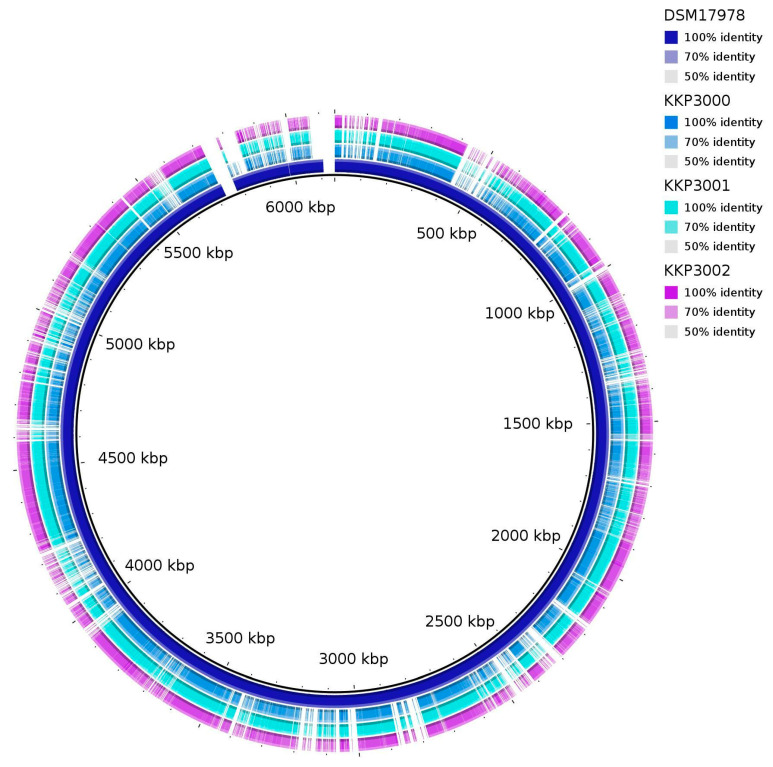
BRIG diagram showing homologous chromosome segments of the three novel strains (KKP 3000, KKP 3001, KKP 3002) to the *A. fastidiosus* type strain DSM 17978.

ANI (Average Nucleotide Identity) analysis between members of the *Alicyclobacillus* spp. and the three novel strains showed that strains KKP 3000, KKP 3001, and KKP 3002 share very high genome similarity (>99%). However, the three strains do not present high ANI with other known *Alicyclobacillus* species. Moreover, the phylogenomic analysis exhibited that the three strains cluster together in a distinct phylogenetic clade, that is closely related to *A. fastidiosus* (Figure 7). The ANI between the three novel strains and members of the *A. fastiodiosus* species was recorded to be 88%, lower than the species cut-off (>96%) (Figure 7). Furthermore, a comparative genetic analysis between the three strains and the type strain *A. fastidiosus* DSM 17978 with BRIG showed distinct genomic differences (Figure 8). Indeed, several regions in the genome of strain DSM 17978 show very low sequence identity (<50%) to the genomic regions of the three novel strains. Based on these findings, further phylogenomic analysis will be pursued to elucidate the taxonomy of the three novel strains.

## 3. Materials and Methods

### 3.1. Bacterial Strains and Growth Conditions

The *Alicyclobacillus* strains used in this study were isolated from Polish pear orchards and pear fruits, as described by Połaska et al. [11] Briefly, the samples were processed according to The International Fruit and Vegetable Juice Association (IFU) Method No. 12, including initial heat shock (80 °C, 10′), enrichment in BAT broth (Merck, Darmstadt, Germany) for 5 days (45 °C), and transferring 10 μL of the enriched culture on the BAT agar (Merck, Darmstadt, Germany). After 3 days of incubation, the obtained colonies were subjected to additional tests, to confirm their affiliation to taint-producing *Alicyclobacillus* group: incubation at 65 °C, guaiacol detection test [41], and erythritol utilization test [42].

All strains, based on the *16S* rDNA identification, were classified as *Alicyclobacillus fastidiosus* and deposited in the Culture Collection of Industrial Microorganisms—Microbiological Resource Center (IAFB, Warsaw, Poland), under collection number KKP 3000, KKP 3001, and KKP 3002. The *16S* rDNA GenBank accession numbers are available for all three strains, KY088044, KY088045, and KY088046.

### 3.2. Genomic DNA Isolation and Sequencing

The total genomic DNA extraction for strain KKP 3000 was performed following the manufacturer protocol with the DNeasy PowerFood Microbial Kit (Qiagen, GmbH, Hilden, Germany), as described by Bucka-Kolendo et al., 2023, and Kiousi et al., 2023 [43,44]. Briefly, the purity of DNA was verified with a Nanodrop ND-1000 Spectrophotometer (Thermo Fisher Scientific, Watertown, MA, USA), and the concentration was measured with a Qubit 4.0 Fluorometer (Qubit dsDNA BR Assay Kit, Invitrogen, Carlsbad, CA, USA). Library preparation and sequencing were performed with the Illumina MiSeq NGS platform (Illumina, San Diego, CA, USA), with an Illumina DNA Prep kit (number #1000000025416v09), the 2 × 151 bp paired-end MiSeq protocol, the 343 reagent v3 (600-cycle) kit. 

For KKP 3001 and KKP 3002, the whole-genome sequencing was commissioned to sequence by GenXone SA (Złotniki, Poland). Libraries were prepared with Rapid Barcoding kit reagents (Oxford Nanopore Technologies, Oxford, UK), with assured 50X genome coverage. WGS was performed in the nanopore technology on the GridlON X5 sequencing device (Oxford Nanopore Technologies, Oxford, UK), with MinKnow v22.10.5 control, as described by Wójcicki et al., 2023 [45] base calling and barcode demultiplexing a Guppy v6.3.8 and Gruppy Barcoder v6.3.8 (Oxford Nanopore Technologies, Oxford, UK) were used. De novo assembly of genomes and annotations were made with Flye v2.8.1. software and Prokka server, respectively. 

### 3.3. Antibiotic Resistance

Nine antibiotics were utilized for Minimum Inhibitory Concentration (MIC) evaluations. The antibiotic resistance profiles of *Alicyclobacillus* spp. strains were assessed through the utilization of the E-test (bioMérieux, Craponne, France) on BAT agar (Merck, Darmstadt, Germany), following the manufacturer’s instructions. E-test strips encompassing a gradient of antibiotics (with a concentration range of 0.016–256 µg/mL) were used and included the following antibiotics: chloramphenicol (catalog no. 412309), clindamycin (catalog no. 412315), erythromycin (catalog no. 412334), gentamicin (catalog no. 412368), kanamycin (catalog no. 412382), streptomycin (catalog no. 526800), and tetracycline (catalog no. 412471), serving as inhibitors of protein synthesis. Additionally, vancomycin (catalog no. 412488) and ampicillin (catalog no. 412253) were used as cell wall synthesis inhibitors.

It is noteworthy that all selected antibiotics are included in the list recommended by the European Food Safety Authority (EFSA) Panel on Additives and Products or Substances used in Animal Feed (FEEDAP) [46]. This list specifies antibiotics against where microbial strains are intended for human use as live culture or probiotics and which animal feed additives should be tested.

The resistance profile of the examined *Alicyclobacillus* strains was established in strict accordance with the manufacturer’s guidelines. After cultivating the strains on BAT agar for 24 h, they underwent centrifugation, followed by two washes with phosphate-buffered saline (PBS). The resulting bacterial pellet was resuspended in PBS buffer with a pH of 7.2, aiming for an optical density of 0.5 McFarland units. Using a swab, the bacterial cultures were evenly applied to BAT agar, covering the entire petri dish. Subsequently, E-tests containing the appropriate antibiotics were placed on the agar dishes inoculated with bacteria. The plates were subsequently incubated under aerobic conditions at 45 °C for 3 days. The MIC results were compared with the reference strain *A. fastidiosus* DSM 17978.

The experiment was carried out in three replicates.

### 3.4. Genome Annotation and Analysis

Genome annotation of the three strains (KKP 3000, KKP 3001, and KKP 3002) was performed with Prokka (version 1.14.5) [47]. EggNOG-mapper (version 2.1.9) and BlastKOALA (version 3) were used for functional annotation of the genomes. Plasmid sequences in the WGS of KKP3000 were investigated with PlasmidFinder [48]. Insertion elements, prophages, and CRISPR arrays were annotated with ISFinder (e-value cut-off: 0.01) [49], PHAge Search Tool Enhanced Release (PHASTER) for prophage regions [50], and CRISPRDetect (version 2.4), and PILER-CR [51], respectively. Acquired antimicrobial resistance genes were identified using Resistance Gene Identifier (RGI; version 5.2.0) and ResFider [51]. Genes coding for virulence factors were annotated with VirulenceFinder 2.0 and PathogenFinder 2.0 [52]. Genome maps were constructed with Proksee, and chromosomes were annotated for antimicrobial resistance genes, mobile elements, and horizontal gene transfer regions with CARD-RGI (version 1.2.0), mobileOG-db (version 1.1.2) and Alien Hunter (version 1.1.0) [53]. The annotated genome of the three strains was manually searched for genes involved in guaiacol production and resistance to erythromycin. To this end, Blastp+ was utilized for the detection of genes encoded by the three novel strains that are homologous to ribosomal RNA small subunit methyltransferase A (RsmA) (WP_021294659.1), benzaldehyde dehydrogenase YfmT (WP_003244104.1), and phenolic acid decarboxylase (WP_003246683.1). Homologous proteins to RsmA were identified using Blastp. The top 24 entries and proteins encoded by the three strains were aligned with ClustalW [54] and produce an unrooted phylogenetic tree was constructed with RAxML. The tree was visualized on the iTol server [55].

### 3.5. Phylogenomic Analysis

The genomic sequences of *A. fastidiosus* DSM 17978 were obtained from the NCBI Assembly database. The Average Nucleotide Identity (ANI) between the three novel strains and the two deposited genomes was calculated with Pyani (version 0.2.10). BLAST Ring Image Generator (BRIG) was used to determine the homology of chromosomal regions of the three novel strains with that of *A. fastidiosus* DSM 17978 (type strain). Phylogenomic trees were constructed based on the whole-genome sequences of the three strains on the TYGS server [56]. Trees were visualized with iTOL [55]. *Phenylobacterium aquaticum* KACC 18306 was used as an outgroup.

## 4. Conclusions

This study determined the antibiotic susceptibility of three novel *Alicyclobacillus* spp. strains isolated from orchard soil and pear both in vitro and in silico. The strains *Alicyclobacillus* spp. KKP 3000, KKP 3001, and KKP 3002 were susceptible to most of the studied antibiotics, excluding erythromycin, to which high resistance was observed. The erythromycin resistance was attributed to the presence of the *rsmA* gene in all three genomes; however, no correlation between antibiotic resistance genes and mobile or transposable elements was found. Furthermore, we identified and characterized the arrangement of the *vdc* operon, which may be involved in the guaiacol production. By analyzing the structure of the operon in the three novel strains and other guaiacol producers, we arrived at the hypothesis that the divergence among the strains may be a result of duplication events that occurred later in time. However, this does not affect their guaiacol production capability. Notably, operons for flagellar assembly and chemotaxis were located, which may be part of the adaptational mechanisms of these strains that will be further explored. Finally, the strains present low Average Nucleotide Identity (ANI) with other members of the *Alicyclobacillus* genus, exhibiting the highest identity to *A. fastidiosus* strains, which was still lower than the species cut-off. Future studies will aim to decipher these novel strains’ evolutionary history and taxonomy.

## Figures and Tables

**Table 1:** Minimum inhibitory concentration (MIC) of selected antibiotics in studied *Alicyclobacillus* strains.

Minimum Inhibition Concentrations Tested [μg/mL]									
Bacteria	AM	GM	KM	SM	EM	CM	TC	CL	VA
KKP 3000	0.094	0.047	0.19	0.5	>256	0.5	0.016	0.5	0.19
KKP 3001	0.064	0.064	0.38	0.5	>256	0.5	0.016	0.75	0.19
KKP 3002	0.047	0.047	0.5	0.5	>256	0.75	0.016	0.75	0.25
*A. fastidiosus* DSM 17978	0.016	0.064	0.5	0.5	>256	0.047	0.016	0.38	0.19

AM—ampicillin, GM—gentamycin, KM—kanamycin, SM—streptomycin, EM—erythromycin, CM—clindamycin, TC—tetracycline, CL—chloramphenicol, VA—vancomycin.

**Table 2:** Genome characteristics of the three *Alicyclobacillus* spp. strains.

Genome Characteristics	*Alicyclobacillus* KKP 3000	*Alicyclobacillus* KKP 3001	*Alicyclobacillus* KKP 3002
Length	4,859,599 bp	4,972,367 bp	4,977,437 bp
GC content	52.95%	53%	52.92%
CDSs	4872	4924	4928
rRNAs	3	45	45
tRNAs	40	131	131
No. of CRISPR Arrays	1	1	1
IS elements	52	14	14
Phages			
Intact	0	0	0
Incomplete	5	7	5
Questionable	0	1	1
Antibiotic resistance genes			
Perfect hits	0	0	0
Strict hits	5	5	5
Loose hits	302	297	297
Virulence genes	0	0	0
Plasmids	0	2	2

**Table 3:** Categorization of genes contained in the genomes of the three strains into clusters of orthologous groups.

Clusters of Orthologous Groups	*Alicyclobacillus* KKP 3000	*Alicyclobacillus* KKP 3001	*Alicyclobacillus* KKP 3002
C-Energy production and conversion	212 (4.96%)	214 (5.5%)	214 (5.5%)
D-Cell cycle control and mitosis	59 (1.38%)	58 (1.49%)	58 (1.49%)
E-Amino Acid metabolism and transport	482 (11.29%)	434 (11.15%)	434 (11,15%)
F-Nucleotide metabolism and transport	99 (2.32%)	99 (2.54%)	99 (2.54%)
G-Carbohydrate metabolism and transport	425 (9.95%)	320 (8.22%)	320 (8.22%)
H-Coenzyme metabolism	183 (4.29%)	163 (4.19%)	163 (4.19%)
I-Lipid metabolism	158 (3.7%)	156 (4.01%)	156 (4.01%)
J-Translation	201 (4.7%)	187 (4.80%)	187 (4.8%)
K-Transcription	374 (8.76%)	352 (9.04%)	352 (9.04%)
L-Replication and repair	218 (5.11%)	265 (6.81%)	268 (6.88%)
M-Cell wall/membrane/envelope biogenesis	217 (5.08%)	193 (4.96%)	193 (4.96%)
N-Cell motility	47 (1.1%)	47 (1.21%)	47 (1.21%)
O-Post-translational modification, protein turnover, chaperone functions	90 (2.11%)	80 (2.06%)	80 (2.05%)
P-Inorganic ion transport and metabolism	278 (6.51%)	201 (5.16%)	200 (5.14%)
Q-Secondary Structure	118 (2.76%)	67 (1.72%)	67 (1.72%)
T-Signal Transduction	151 (3.54%)	111 (2.85%)	111 (2.85%)
U-Intracellular trafficking and secretion	49 (1.15%)	31 (0.80%)	31 (0.8%)
V-Defence mechanisms	56 (1.31%)	58 (1.49%)	58 (1.49%)
S-Function Unknown	696 (16.3%)	697 (17.91%)	696 (17.9%)
No annotation	157 (3.68%)	159 (4.09%)	159 (4.09%)
Total (%)	4270 (100%)	3892 (100%)	3893 (100%)

**Table 4:** Annotation of genes in the genomes of KPP 3000, KKP 3001, and KKP 3002 involved in sporulation, germination, flagellar assembly, and chemotaxis.

*Alicyclobacillus* KKP 3000	*Alicyclobacillus* KKP 3001	*Alicyclobacillus* KKP 3002	Predicted Protein	*Gene*
**Sporulation and Germination**				
KFAPOJEI_02576	PLKKBLCC_02372	PKEAFELD_02372	stage III sporulation protein AG	*spoIIIAG*
KFAPOJEI_02575	PLKKBLCC_02373	PKEAFELD_02373	Stage III sporulation protein AF	*spoIIIAF*
KFAPOJEI_02574	PLKKBLCC_02374	PKEAFELD_02374	stage III sporulation protein AE	*spoIIIAE*
KFAPOJEI_02573	PLKKBLCC_02375	PKEAFELD_02375	Stage III sporulation protein AD	*spoIIIAD*
KFAPOJEI_02572	PLKKBLCC_02376	PKEAFELD_02376	stage III sporulation protein AC	*spoIIIAC*
KFAPOJEI_02571	PLKKBLCC_02377	PKEAFELD_02377	Stage III sporulation protein AB	*spoIIIAB*
KFAPOJEI_02570	PLKKBLCC_02378	PKEAFELD_02378	stage III sporulation protein AA	*spoIIIAA*
KFAPOJEI_03030	PLKKBLCC_01460	PKEAFELD_01459	Sporulation sigma-E factor-processing peptidase	*spoIIGA*
KFAPOJEI_02435	PLKKBLCC_04809	PKEAFELD_04813	Stage II sporulation protein R	*spoIIR*
KFAPOJEI_01887	PLKKBLCC_04452	PKEAFELD_04456	Spore cortex biosynthesis protein YabQ	*yabQ*
KFAPOJEI_01888	PLKKBLCC_04453	PKEAFELD_04457	Spore coat protein	*yabP*
KFAPOJEI_02376	PLKKBLCC_04750	PKEAFELD_04754	Spore Coat Protein X and V domain	*cotX*
KFAPOJEI_02378	PLKKBLCC_04752	PKEAFELD_04756	Spore Coat Protein X and V domain	*cotX*
KFAPOJEI_02939	PLKKBLCC_01782	PKEAFELD_01782	Outer spore coat protein E	*cotE*
KFAPOJEI_03520	PLKKBLCC_03012	PKEAFELD_03013	Spore coat associated protein JA	*cotJA*
KFAPOJEI_03586	PLKKBLCC_01820	PKEAFELD_01821	Bacillus/Clostridium GerA spore germination protein	*spoVAF*
KFAPOJEI_02916	PLKKBLCC_01759	PKEAFELD_01759	Dipicolinate synthase subunit B	*spoVFB*
KFAPOJEI_00796	PLKKBLCC_02257	PKEAFELD_02257	Stage IV sporulation protein A	*spoIVA*
KFAPOJEI_00087	PLKKBLCC_03559	PKEAFELD_03564	Spore germination protein	*gerBA*
KFAPOJEI_03728	PLKKBLCC_04348	PKEAFELD_04352	Spore gernimation protein	*gerD*
KFAPOJEI_01384	PLKKBLCC_02819	-	Germination protease	*gpr*
KFAPOJEI_00085	PLKKBLCC_03561	PKEAFELD_03566	PFAM spore germination B3 GerAC family protein	*yfkR*
KFAPOJEI_02645	PLKKBLCC_02303	PKEAFELD_02303	Sporulation protein YtfJ	*ytfJ*
KFAPOJEI_02649	PLKKBLCC_02299	PKEAFELD_02299	Sporulation lipoprotein YhcN/YlaJ	*yhcN*
KFAPOJEI_00243	PLKKBLCC_03402	PKEAFELD_03408	Small, acid-soluble spore protein D	*sspD*
KFAPOJEI_01029	PLKKBLCC_03882	PKEAFELD_03887	Small, acid-soluble spore protein Tlp	*tlp*
KFAPOJEI_02647	PLKKBLCC_02301	PKEAFELD_02301	Spore maturation protein B	*spmB*
**Flagella formation and Chemotaxis**				
KFAPOJEI_02858	PKEAFELD_01701	PLKKBLCC_01701	Flagellar basal body rod protein FlgB	*flgB*
KFAPOJEI_02859	PKEAFELD_01702	PLKKBLCC_01702	Flagellar basal-body rod protein FlgC	*flgC*
KFAPOJEI_02860	PKEAFELD_01703	PLKKBLCC_01703	Flagellar hook-basal body complex protein FliE	*fliE*
KFAPOJEI_02861	PKEAFELD_01704	PLKKBLCC_01704	Flagellar M-ring protein	*fliF*
KFAPOJEI_02862	PKEAFELD_01705	PLKKBLCC_01705	Flagellar motor switch protein FliG	*fliG*
KFAPOJEI_02863	PKEAFELD_01706	PLKKBLCC_01706	Flagellar assembly protein FliH	*fliH*
KFAPOJEI_02864	PKEAFELD_01707	PLKKBLCC_01707	Flagellum-specific ATP synthase	*fliI*
KFAPOJEI_02866	PKEAFELD_01709	PLKKBLCC_01709	Flagellar protein FlbB	*flbB*
KFAPOJEI_02868	PKEAFELD_01711	PLKKBLCC_01711	Minor extracellular protease Vpr	*flgD*
KFAPOJEI_02870	PKEAFELD_01713	PLKKBLCC_01713	Flagellar basal-body rod protein FlgG	*flgG*
KFAPOJEI_02871	PKEAFELD_01714	PLKKBLCC_01714	Flagellar protein FlbD	*flbD*
KFAPOJEI_02872	PKEAFELD_01715	PLKKBLCC_01715	Flagellar protein FliL	*fliL*
KFAPOJEI_02873	PKEAFELD_01716	PLKKBLCC_01716	Flagellar motor switch protein FliM	*fliM*
KFAPOJEI_02874	PKEAFELD_01717	PLKKBLCC_01717	Flagellar motor switch protein FliN	*fliY*
KFAPOJEI_02875	PKEAFELD_01718	PLKKBLCC_01718	Chemotaxis protein CheY	*cheY*
KFAPOJEI_02876	PKEAFELD_01719	PLKKBLCC_01719	Flagellar biosynthesis protein FliO	*fliO*
KFAPOJEI_02877	PKEAFELD_01720	PLKKBLCC_01720	Flagellar biosynthetic protein FliP	*fliP*
KFAPOJEI_02878	PKEAFELD_01721	PLKKBLCC_01721	Flagellar biosynthetic protein FliQ	*fliQ*
KFAPOJEI_02879	PKEAFELD_01722	PLKKBLCC_01722	Flagellar biosynthetic protein FliR	*fliR*
KFAPOJEI_02880	PKEAFELD_01723	PLKKBLCC_01723	Flagellar biosynthetic protein FlhB	*flhB*
KFAPOJEI_02881	PKEAFELD_01724	PLKKBLCC_01724	Flagellar biosynthesis protein FlhA	*flhA*
KFAPOJEI_01101	PKEAFELD_03959	PLKKBLCC_03954	Pilus assembly protein PilZ	*pilZ*
KFAPOJEI_04448	PKEAFELD_04051	PLKKBLCC_04046	Transcriptional regulator of flagella formation YvyF	*yvyF*
KFAPOJEI_04452	PKEAFELD_04047	PLKKBLCC_04042	Flagellar hook-associated protein 1	*flgK*
KFAPOJEI_04453	PKEAFELD_04046	PLKKBLCC_04041	Flagellar hook-associated protein 3	*flgL*
KFAPOJEI_04454	PKEAFELD_04045	PLKKBLCC_04040	Flagellin	*fliC*
KFAPOJEI_04455	PKEAFELD_04044	PLKKBLCC_04039	Flagellar hook-associated protein 2	*fliD*
KFAPOJEI_04457	PKEAFELD_04042	PLKKBLCC_04037	Flagellar secretion chaperone FliS	*fliS*
KFAPOJEI_01101	PKEAFELD_03959	PLKKBLCC_02269	Pilus assembly protein PilZ	*pilZ*
KFAPOJEI_03756	PKEAFELD_00013	PLKKBLCC_00013	Motility protein A	*motA*
KFAPOJEI_03757	PKEAFELD_00014	PLKKBLCC_00014	Motility protein B	*motB*
PKEAFELD_02776	PKEAFELD_02776	PLKKBLCC_02776	Putative sensory transducer protein YfmS	*yfmS*
PKEAFELD_00239	PKEAFELD_00239	PLKKBLCC_00239	Inhibitor of MCP methylation, homolog of CheC	*cheX*
PKEAFELD_01727	PKEAFELD_01727	PLKKBLCC_01727	CheB methylesterase	*cheB*
PKEAFELD_01728	PKEAFELD_01728	PLKKBLCC_01728	Signal transducing histidine kinase, homodimeric domain	*cheA*
PKEAFELD_01729	PKEAFELD_01729	PLKKBLCC_01729	Two component signalling adaptor domain	*cheW*
PKEAFELD_01730	PKEAFELD_01730	PLKKBLCC_01730	CheY-P phosphatase CheC	*cheC*
PKEAFELD_01731	PKEAFELD_01731	PLKKBLCC_01731	Chemoreceptor glutamine deamidase CheD	*cheD*
PKEAFELD_02249	PKEAFELD_02249	PLKKBLCC_02249	Methyltransferase, chemotaxis proteins	*cheR*

## Data Availability

The *Alicyclobacillus* strain KKP 3000, KKP 3001, and KKP 3002 genome sequences have been deposited at DDBJ/ENA/GenBank under the accessions SAMN41503782, SAMN41503783, and SAMN41503784, respectively.

## Supplementary Materials

The following supporting information can be downloaded at: https://www.mdpi.com/article/10.3390/ijms25158144/s1. References [57,58] are cited in Supplementary Materials.
